# Electronic screen technology use and connection to nature in Canadian adolescents: a mixed methods study

**DOI:** 10.17269/s41997-019-00289-y

**Published:** 2020-02-05

**Authors:** Valerie Michaelson, Nathan King, Ian Janssen, Sabreena Lawal, William Pickett

**Affiliations:** 1 Department of Health Sciences, Brock University, St. Catharines, ON Canada; 2 Department of Public Health Sciences, Queen’s University, Kingston, ON Canada; 3 School of Kinesiology and Health Studies, Queen’s University, Kingston, ON Canada; 4 School of Medicine, Queen’s University, Kingston, ON Canada

**Keywords:** Adolescence, Child, Electronic screens, Epidemiology, Mixed methods, Nature, Qualitative research, Technology, Adolescence, Enfant, Écrans électroniques, Épidémiologie, Méthodes mixtes, Nature, Recherche qualitative, Technologie

## Abstract

**Objectives:**

Declines in exposure to nature may deprive young people of experiences that are positive for their mental health. One factor that may interfere with connections to nature is use of electronic screen technologies. The objectives of this study are to (1) document variations in the perceived importance of connections to nature nationally among adolescents; (2) explore relationships between these connections and the use of electronic screens, both epidemiologically and qualitatively; and (3) integrate core findings from both strands in order to provide evidence-based recommendations for health promotion.

**Methods:**

The study involved a mixed methods design. Strand 1 involved a qualitative study of 74 Canadians (ages 10–18, years 2016–2018) with data collected through focus groups and interviews. Strand 2 involved a cross-sectional observational analysis of a national survey of 23,920 Canadians (ages 11–15, years 2013–2014). Findings from both strands were integrated through an established protocol.

**Results:**

Increased use of electronic screen technology was consistently associated with lower perceived importance of connections to nature. Barriers to connecting to nature included choices that young people are making, the addictive properties of technology, and beliefs that being indoors is more comfortable and safer than being outdoors. When young people disconnected, their appreciation of being outdoors increased.

**Interpretation:**

This novel study showed, quantitatively, that the time young people spend with electronic screens displaces time that they spend engaging in outdoor activities. Deeper reasons why such associations occur emerged in the qualitative strand. Temporary disconnection from screens may lead to renewed opportunities for outdoor exposures.

## Résumé

**Objectifs:**

La baisse de l’exposition à la nature peut priver les jeunes d’expériences bénéfiques pour leur santé mentale. L’un des facteurs qui peut brouiller leurs contacts avec la nature est l’usage des écrans électroniques. Notre étude vise à : 1) rendre compte des écarts dans l’importance perçue des contacts avec la nature, à l’échelle nationale, chez les adolescents; 2) explorer les relations entre ces contacts et l’usage des écrans électroniques, tant sur le plan épidémiologique que qualitatif; et 3) intégrer les constatations de base des deux volets pour formuler des recommandations factuelles à des fins de promotion de la santé.

**Méthode:**

Il s’agit d’une étude à méthodes mixtes. Le premier volet était une étude qualitative auprès de 74 Canadiens (âgés de 10 à 18 ans, 2016-2018) avec des données collectées lors de groupes thématiques et d’entretiens. Le deuxième volet a consisté en l’analyse observationnelle transversale d’une enquête nationale auprès de 23 920 Canadiens (âgés de 11 à 15 ans, 2013-2014). Les constatations des deux volets ont été intégrées au moyen d’un protocole établi.

**Résultats:**

L’usage accru des écrans électroniques était systématiquement associé à une moindre importance perçue des contacts avec la nature. Les obstacles au contact avec la nature étaient les choix faits par les jeunes, les effets d’accoutumance de la technologie et la conviction qu’il est plus confortable et moins risqué d’être à l’intérieur qu’à l’extérieur. Quand les jeunes se « débranchaient », leur appréciation du temps passé dehors augmentait.

**Interprétation:**

Cette étude novatrice montre, en chiffres, que le temps passé par les jeunes devant des écrans électroniques remplace le temps qu’ils consacrent aux activités de plein air. Les raisons profondes de telles associations sont apparues dans le volet qualitatif. La déconnexion temporaire des écrans peut raviver les possibilités de s’exposer à la nature.

## Introduction

Despite opportunities that young people have to be exposed to the outdoors and natural world, exposures to nature are on the decline, including among children and youth (Louv 2016). Such declines may deprive young people of the potential benefits of engagement with nature, be they physical, spiritual, mental or social dimensions of health and well-being (Kuo et al. 2019; Brooks et al. 2018, Piccininni et al. 2018). Nature refers to products of the earth’s physical world, including the landscape and plants, as opposed to humans and human creations. A recent systematic review of 35 studies reported that exposure to nature positively influences several aspects of mental health within children and youth, including emotional well-being, self-esteem, quality of life, and symptoms of depression and attention deficit disorder (Tillmann et al. 2018).

Explanations as to why young people are being exposed to nature less frequently could involve “structural” (e.g., socio-economic and political mechanisms that drive health inequities) or “intermediate” (behavioural and psychological factors that lead to health inequalities) determinants (Solar and Irwin 2010). However, few large-scale studies have provided in-depth examination of the etiology of this phenomenon. Existing studies have typically been focused on adult populations (e.g., Maas et al. 2009; Van den Berg et al. 2015), and they have employed assessments of the amount of natural space in participants’ neighbourhoods as primary indicators of exposure to nature (Huynh et al. 2013; Kyttä et al. 2012). Barriers that prevent young people from being exposed to nature therefore remain incompletely understood.

One compelling hypothesis for the observed declines in exposure to nature among young people is that high screen-time levels are in part responsible for declines in the importance of nature in the lives of young people (Tremblay et al. 2015). Such practices may, intentionally or unintentionally, interfere with efforts to connect with natural settings and contexts. Given the well-established value of nature to the health of young people, development of new evidence that critically examines this hypothesis is warranted. This could bolster the content of interventions and health promotion strategies that focus intentionally on exposures to nature as a positive health asset. Elimination of or managing the barriers that prevent exposures to nature in children represent potential public health strategies that may be protective, accessible and affordable on a population basis (Davison et al. 2011).

In order to address this gap in knowledge, we developed a national study that employed a mixed methods strategy in which we integrated both quantitative and qualitative strands of data collections and analyses. This was done in order to (1) document variations in the perceived importance of connections to nature nationally among adolescents; (2) explore relationships between these connections and the use of electronic screens, both epidemiologically and qualitatively, the latter based upon the lived experiences of adolescent boys and girls; and (3) interpret and synthesize core findings from both strands in order to provide evidence-based recommendations for prevention and health promotion. Typical assessments in the past have measured exposure to nature as the amount of natural space in a respondents’ neighbourhood (e.g., how much green space is within 1 km of a youth’s home address). However, availability of natural space may represent a bad proxy for the time that a young person spends in nature and their connection to nature. Through our study, we hoped to advance this field of research through the assessment of youths’ deep connections to nature (beyond living in a neighbourhood that has green space).

## Methods

### Mixed methods design

**Fig. 1:**
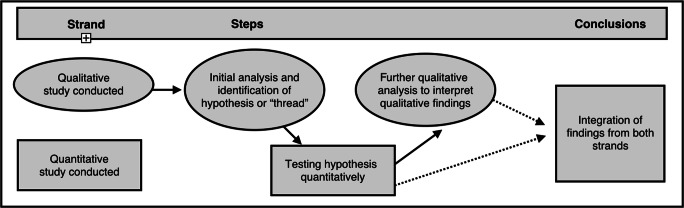
Schema describing integrated mixed methods design

We employed a sequential mixed methods study design (Fig. 1), with the intention that it would provide us with a more comprehensive understanding of our research question than either method in isolation would yield (Clark and Cresswell, 2008). In strand 1, we employed a qualitative study that was guided by Thorne’s approach to interpretive description (*n* = 74). Strand 2 involved a cross-sectional observational analysis of a national survey (Freeman 2016) of 23,920 young Canadians aged 11–15 years. Our initial analysis of qualitative (strand 1) observations generated a hypothesis or “thread” (Moran-Ellis et al. 2006) for testing in our quantitative data (strand 2). Following this testing, we ultimately followed this “thread” back to the qualitative data set to help interpret the quantitative findings. Final inferences were summarized thematically based on the results of both strands of enquiry.

### Ethics

Strand 1 of our study was approved by the Queen’s University Health Sciences and Affiliated Teaching Hospitals Research Ethics Board (February 2016). Strand 2 was approved by the General Research Ethics Board at Queen’s University (May 2017) and the Health Canada / Public Health Agency of Canada Research Ethics Board (May 2016).

### Qualitative strand (strand 1)

#### Participants

Participants were recruited as part of a study that was conducted across Canada in 2016–2018, with an initial primary focus on understanding the spiritual health of young people, one domain of which is the importance of “connections to nature” (Michaelson et al. 2016a, Michaelson et al. 2016b, Brooks et al. 2018, Piccininni et al. 2018, Michaelson et al. 2019). A purposeful, criterion-based approach was used for recruitment, with criteria of interest being age, gender, immigration status, urban-rural status and religious/spiritual/cultural backgrounds, including atheist and agnostic self-identification. Our final sample included 74 young people who were primarily aged 11–15 years, with several outliers to make the complete sample 10–18 years. Data collection took place in the Lower Mainland of British Columbia, Central Alberta, Northern Ontario, a large urban centre in Ontario, Eastern Ontario, the Eastern Townships in Quebec, Northern Canada, Prince Edward Island, and a city in Nova Scotia.

Data were collected through 7 focus groups and 21 semi-structured interviews. For focus groups, participants who shared one or more criteria (including age, gender, rural/urban geographic status, and immigration status) were recruited in order to form homogenous groups that would facilitate conversation. Participants were recruited using “snowball” or “chain” sampling. Letters of information and informed consent were given to well-situated people, such as leaders of youth community organizations (e.g., Boys and Girls clubs) and educators, who we predicted might be aware of potential participants in seven communities from across Canada. These people assisted us by circulating the study information in their community.

#### Data collection

To invite rich discussion, the focus groups and interviews involved core, open-ended questions that were asked of each group or person. These initially asked participants to describe their understanding of the importance in their lives of four standard domains describing the broader concept of spiritual health (connections to self, others, nature, and to some kind of experiences of the transcendent or larger meaning to life) (Michaelson et al. 2016a, Michaelson et al. 2016b, Brooks et al. 2018, Piccininni et al. 2018, Michaelson et al. 2019; Gomez and Fisher 2003; Hay and Nye 2006) and how each related to the mental health status of themselves and other young people. In the current secondary analysis, we specifically focused in on the connections to nature domain.

#### Analysis

Focus groups and interviews were transcribed verbatim and examined consecutively line-by-line in order to identify descriptions of thought patterns, feelings and actions that emerged throughout. Coding was conducted by two investigators (VM and SL). Codes were formulated from emergent observations, and were then compared to verify their descriptive content and to confirm that they were rooted in the data. A second level of axial coding was then applied, which resulted in the identification of higher-level, more conceptual categories. To avoid confusion between strands, we refer to these categories as “observations” and reserve the more conventional term “themes” for the inferences that emerged upon integration with the quantitative stage. At the third level of coding, all authors (VM, SL, WP, IJ and NK) were consulted in order to further enhance the rigour of the coding and associated analytic process.

Triangulation among all authors was employed during the entire process of analysis. Investigators were intentional in identifying their own biases in relation to the research question, and they discussed these in order to make visible unconscious bias. Again, all of the authors were involved with discussing and comparing codes and categories in order to provide multiple interpretive perspectives and so to improve rigour. The rich, thick description that emerged within the entire data set also enhanced the credibility of our findings (Patton 2005).

### Quantitative strand (strand 2)

#### Participants

The quantitative strand involved a cross-sectional observational analysis of the nationally representative 2013–2014 Canadian Health Behaviour in School-aged Children (HBSC) study (Freeman 2016) of 23,920 young Canadians primarily aged 11 to 15 years. As per our qualitative sample, there were younger and older outliers to this range. The HBSC study involved a standardized protocol, targeted to the pre- and early adolescent years, and involved completion of written health surveys in classroom settings. The sample was stratified by province/territory, type of school board (public vs. separate), urban-rural geographic status, school population size, and language of instruction (French vs. English) (Freeman 2016). Standardized population weights were generated to account for sampling differences by grade when compared with underlying provincial/territorial distributions. Exclusions were youth from private schools, home schools, First Nations reserves, street youth, incarcerated youth, youth otherwise absent on the day of the survey, or youth not providing informed consent.

### Measurements

#### Exposures to electronic screens

Respondents to the HBSC were categorized according to items that assessed the typical time (on weekdays and weekends) spent (1) watching TV programs, videos and movies; (2) playing video games; and (3) using a computer for other purposes (e.g., homework, emailing, tweeting, Facebook, chatting, surfing the Internet) (McMillan et al. 2015). We summed the reported amounts of exposure to these items to get an estimate of average daily time on electronic screen technology. We also categorized responses into different categories of use. Finally, in order to capture the extent of use of specific types of technology for communication purposes, we identified daily vs. non-daily use of the following: (1) texting/SMS; (2) social media; and (3) instant messaging to contact their friends.

#### Connections to nature

A single item from the HBSC spiritual health module (Michaelson et al. 2016a, Michaelson et al. 2016b, Brooks et al. 2018, Piccininni et al. 2018, Michaelson et al. 2019) (adapted from Fisher’s Spiritual Well-being Scale for Secondary Students) (Gomez and Fisher 2003) was used to estimate the importance of exposures to nature in the lives of adolescents. This adapted spiritual health module itself consisted of 8 items in four standard domains describing the importance of connections to self, others, nature, and the transcendent. The adapted module was piloted both qualitatively and quantitatively, and based on findings from two countries, it was revised to foster understanding and to serve the literacy needs of children as young as 11 years of age. Exploratory factor analyses demonstrated that the 8 items could be combined into a unidimensional scale (*α* = 0.80), although subsequent confirmatory factor analysis suggested that analyses best be conducted by domain or with the 8 individual items (Michaelson et al. 2016a). For one of these items, respondents were requested to identify how important it was for them to “feel connected to nature” using five response categories ranging from 1—“not at all important” to 5—“very important”. Based on precedent (Michaelson et al. 2016a), scores of 4 or 5 were rated as “important”.

#### Demographics

Students reported their gender (“boy” or “girl”). Based on reported birth years and months and the dates of survey administration, age in years was estimated and categorized into three groups (“≤ 12”, “13–14”, “≥ 15” years). Based on school postal codes and a Statistics Canada coding system (Statistics Canada 2016), we also classified each student according to their geographic status: “rural” (rural or small centres) vs. “urban” (medium, large or metropolitan centres).

#### Data analysis

We estimated the percentages of young people who rated that connections to nature were “important” in their lives, overall and by age and gender. We next profiled the daily amounts of exposure to various electronic screen technologies (mean and SD, then percent reporting the highest levels of use). We also estimated the percentage of young people reporting daily use of texting/SMS, social media use, and instant messaging to contact their friends. We used standard population weights to ensure that findings were nationally representative. We then tested an initial hypothesis or “thread” (Moran-Ellis et al. 2006) that emerged both qualitatively and in background literature (Tremblay et al. 2015)—that high screen-time levels are in part responsible for declines in the importance of nature in the lives of young people. We used graphical analyses to illustrate trends and patterns descriptively. Log-binomial regression models, which accounted for clustering by schools, were used to evaluate statistical tests for trends across categories of daily exposures to electronic screen technology and also potential interactions by age and gender. Similar analyses were used to examine associations between frequency (less than weekly, weekly, daily) of texting, social media use, and instant messaging and the reported importance of nature in the lives of respondents. All data analyses were conducted with SAS 9.4 (Cary, NC). A list of quantitative “observations” was then created, to be integrated with those from the qualitative strand.

### Mixed methods integration

#### Integration strategy 1: “following a thread”

The first integration strategy, as recommended by O’Cathain et al. (2010) and described by Moran-Ellis et al. (2006), involved “following a thread”. Initial results from strand 1 were used to form hypotheses that could be explored in strand 2, and vice versa. Following identification of the thread in strand 1 (qualitative), we therefore began with an analysis of the quantitative data set to explore the idea that exposures to electronic screen technology and the importance of connections to nature were related. This enabled us to identify whether or not an “observation” in one strand could be followed iteratively between both strands (“the thread” (Moran-Ellis et al. 2006)). In this way, we hoped to “create a constellation of findings which [could] be used to generate a multi-faceted picture of the phenomenon” (Moran-Ellis et al. 2006, p. 54). This integration strategy “preserves the value of the open, exploratory, qualitative inquiry” but also “incorporate[s] the focus and specificity of the quantitative data” (Moran-Ellis et al. 2006, page 54).

#### Integration strategy 2: triangulation protocol

The second integration strategy followed a “triangulation protocol” developed by Farmer et al. (2006) and recommended by O’Cathain et al. (2010). Six steps are involved in this protocol: (1) Identification of the key “observations” from both methodological strands; (2) convergence coding (determining the level of agreement between two strands); (3) convergence assessment (a global assessment of convergence of all strands); (4) completeness assessment (comparing the scope of all unique topic areas that were present in one strand and silent in the other); (5) researcher comparison (documenting where researchers had different opinions about convergence); and (6) feedback (reviewing triangulated results with stakeholders).

Four investigators (VM, IJ, NK, WP) applied this protocol and considered whether there was full agreement, partial agreement, “silence” (one strand provided no data), or “dissonance” (observations from the strands disagreed) between observations from the qualitative and quantitative strands. This process enabled us as researchers to move from thinking about the findings related to each method in isolation, to findings based on an integrated whole. Systematically comparing and contrasting findings from the two strands allowed us to see if understanding about our topic of interest was enhanced. It also helped us to consider the possibility of themes surrounding the study “thread” that transcended both strands of the study.

## Results

### Participant characteristics

The qualitative sample included 74 young people aged 10–18 years. Data were collected through 7 focus groups ranging in size from 4 to 9 (median 6) participants, with an additional 21 semi-structured interviews. The quantitative sample was drawn from 30,153 young people within the target age range of 11–15 years who participated in the 2013–2014 Canadian HBSC, representing a response rate of 77% at the individual student level. For this analysis, we had complete information on 23,920 young people (11,377 boys, 12,543 girls; weighted *n* = 11,376 boys, 12,647 girls).

### The “thread”

Qualitative analyses of early focus groups and interviews led to the identification of an emergent hypothesis or thread that *high screen-time levels are in part responsible for declines in the importance of nature in the lives of young people.*

### Descriptive observations

*Quantitatively*, 60.4% of boys and 62.7% of girls reported that feeling connected to nature was important (score ≥ 4 of 5) in their lives. These percentages decreased with age in both genders (boys 68.3%, 60.2% and 54.1% for ages ≤ 12, 13–14 and ≥ 15, respectively; girls 72.7%, 60.9% and 56.1%). Among both boys and girls, we observed consistent associations that indicated that as age increased, the use of all forms of electronic screen technology also increased (Table 1). Girls in all age groups reported more frequent exposures to daily texting/SMS, social media use, and instant messaging in order to communicate with friends, as well as high levels of other computer use. Boys reported more frequent exposures to video game use and watching TV programs, movies, and videos.

**Table 1:** Percentage of young Canadians reporting exposures to electronic screen technology, 2014 HBSC Study (weighted *n* = 24,023)

	Age group			
	Full sample	≤ 12	13–14	≥ 15
Boys	*n* = 11,376	*n* = 3014	*n* = 4718	*n* = 3644
Mean (SD) daily hours of exposure				
Total	7.7 (4.7)	6.5 (4.4)	7.9 (4.6)	8.5 (4.9)
Watching TV, movies, videos	2.7 (1.8)	2.4 (1.7)	2.7 (1.8)	2.8 (1.9)
Playing video games	2.7 (2.0)	2.3 (1.8)	2.8 (2.0)	2.8 (2.1)
Other computer use	2.4 (2.0)	1.8 (1.8)	2.4 (2.0)	2.8 (2.2)
No. (%) with high levels of exposure				
Total screen time > 9 h/day	3713 (32.6)	722 (24.0)	1560 (33.9)	1392 (38.2)
Watching TV, movies, videos >4 h/day	2284 (20.1)	471 (15.6)	970 (20.6)	844 (23.2)
Playing video games >4 h/day	2673 (23.5)	549 (18.2)	1151 (24.4)	973 (26.7)
Other computer use >4 h/day	2280 (20.1)	376 (12.5)	992 (21.0)	913 (25.1)
No. (%) reporting daily use				
Texting/SMS	3937 (34.6)	639 (21.2)	1655 (35.1)	1642 (45.1)
Social media	3106 (27.3)	610 (20.2)	1374 (29.1)	1122 (30.8)
Instant messaging	4197 (36.9)	711 (23.6)	1835 (38.9)	1651 (45.3)
Girls	*n* = 12,647	*n* = 3374	*n* = 5358	*n* = 3915
Mean (SD) daily hours of exposure				
Total	7.5 (4.7)	6.1 (4.5)	7.9 (4.7)	8.2 (4.7)
Watching TV, movies, videos	2.5 (1.7)	2.2 (1.7)	2.6 (1.7)	2.6 (1.7)
Playing video games	2.1 (2.1)	1.9 (1.8)	2.2 (2.1)	2.2 (2.2)
Other computer use	2.9 (2.2)	2.0 (1.9)	3.1 (2.2)	3.4 (2.2)
No. (%) with high levels of exposure				
Total screen time > 9 h/day	4132 (32.7)	742 (22.0)	1870 (34.9)	1520 (38.8)
Watching TV, videos, movies >4 h/day	2283 (18.1)	480 (14.2)	1008 (18.8)	794 (20.3)
Playing video games >4 h/day	2375 (18.8)	495 (14.7)	1078 (20.1)	802 (20.5)
Other computer use >4 h/day	3586 (28.4)	518 (15.4)	1670 (31.2)	1398 (35.7)
No. (%) reporting daily use				
Texting/SMS	7023 (55.5)	1316 (39.0)	3091 (57.7)	2616 (66.8)
Social media	4720 (37.3)	933 (27.6)	2153 (40.2)	1635 (41.8)
Instant messaging	6919 (54.7)	1338 (39.7)	3200 (59.7)	2380 (60.8)

### Etiological observations

**Fig. 2:**
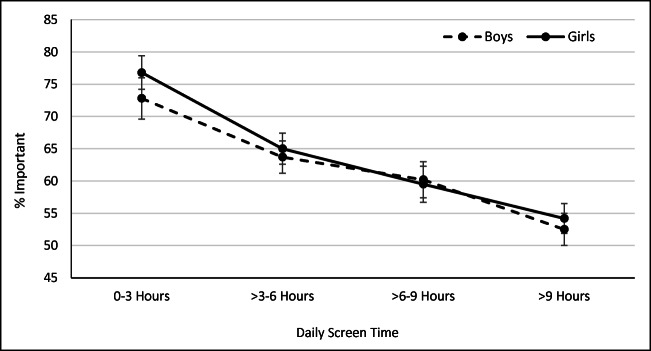
Percentage of young people rating connections to nature as important by daily average hours of electronic screen technology use, by gender; 2014 HBSC Sample, weighted *n* = 11,376 boys and 12,647 girls. Values are weighted. Test for interaction, *p* value = 0.49. Tests for trend: boys, *p* trend < 0.0001; girls, *p* trend < 0.0001

**Fig. 3:**
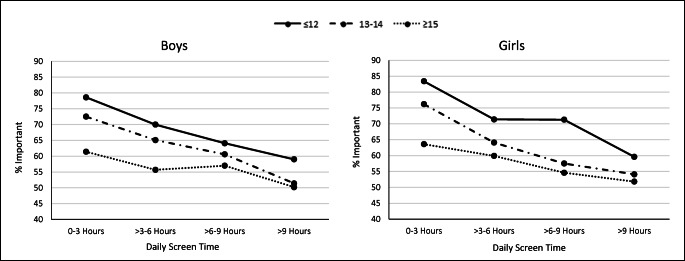
Percentage of young people rating connections to nature as important by average daily hours of electronic screen technology use, by age group for boys and girls; 2014 HBSC Sample, weighted *n* for boys ≤ 12 = 3014; boys 13–14 = 4718; boys ≥ 15 = 3644; girls ≤ 12 = 3374; girls 13–14 = 5358; girls ≥ 15 = 3915. Values are weighted. Tests for interaction by age group: boys *p* value = 0.08; girls *p* value = 0.11. Tests for trend: boys ≤ 12, *p* trend = 0.001; boys 13–14, *p* trend < 0.001; boys ≥ 15, *p* trend = 0.02; girls ≤ 12, *p* trend < 0.001; girls 13–14, *p* trend < 0.001; girls ≥ 15, *p* trend < 0.001

**Fig. 4:**
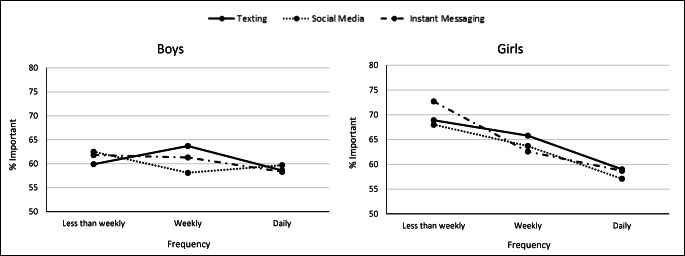
Percentage of young people rating connections to nature as important by frequency of texting, social media use, and instant messaging (e.g., Facebook chat) to talk to friends, for boys and girls; 2014 HBSC Sample, weighted *n* for boys = 11,376; girls = 12,647. Values are weighted. Tests for trend: boys, texting, *p* trend = 0.73; boys, social media, *p* trend = 0.14; boys, instant messaging, *p* trend = 0.09; girls, texting, *p* trend < 0.001; girls, social media, *p* trend < 0.001; girls, instant messaging, *p* trend < 0.001

*Quantitatively*, among boys and girls, as the daily hours of electronic screen technology increased, there was a corresponding decrease in the reporting that connections to nature were important (Fig. 2). These relationships were each strong and statistically significant (*p* trend < 0.001), with no evidence of an interaction by gender (*p* = 0.49). Figure 3 describes similar relationships, but this time stratifying by gender and age group. Again, as the daily hours of electronic screen technology increased, there was a corresponding decrease in reporting that connections to nature were important in all six strata defined by age and gender. These associations were particularly strong in young children. However, when we examined the frequency of exposure to texting, social media use, and instant messaging, we identified strong relationships between electronic communication use and declines in the importance of nature among girls, while exposures to electronic communication had little bearing on the importance of nature among boys (Fig. 4).

*Qualitatively*, when we followed the “thread” back, we found that irrespective of age, gender, geography or background, young people interviewed from across the country consistently reported that use of electronic screen technology distances them from nature. Some of this phenomenon relates to the very distinct choices that young people are making about ensuring that ongoing use of electronic screens is possible and a priority in their lives. We have organized their responses into four related “observations”.

1. Many young people prioritize being indoors accessing electronic screens over being outdoors in nature

Young people from across Canada are choosing to use electronic screens often and in many and varied forms. For many, this is a personal and inherently social choice. One unintended cost of this reliance on electronic screens is the competing risk of lost opportunity to be exposed to nature. Very practically, screens are often best engaged with indoors. These two quotes are examples of what we were told by participants with respect to this observation:

“Well I feel the biggest reason [young people are less connected to nature] is technology. And so instead of being outside kids are inside playing video games and on their phones and texting friends. Just in general they are on technology and inside. And I feel that is the biggest reason why more people are not outside.” Female, age 13

“I like the environment. [My friends] don’t like it. They don’t like to be outside. They like to be on their electronics, Sometimes [your phone] distracts you from your school work and from going outside and having fun and not being in the house all day on your phone....and yeah.” Male, age 11

Observation 2. Screen-time technology and its addictive properties interfere with connections to nature

A related but separate observation that emerged is the addictive properties of electronic screens and communication technology, and consequent demands on the time and attention of young people. Whether it is a perceived need to be in continual contact with peers or family by text or message, or to keep a “streak” alive on an online platform, or to be playing video games competitively, the use of screens can be addictive. We were told that while it is possible to limit one’s time on electronic screen technology, most people do not, and instead get “caught up” in it:

“I am sure [screens and other technologies] be used well and you can limit your time on them and use it for good. But most of the time people don’t and it takes away from being outside or what is happening in the real world or other places.” Male, age 15

“I think people get so caught up in social media and stuff like Snap Chat that they don’t realize that connecting to nature helps you cope with the stress you have other than just going on your phone or hanging out with friends.” Female, age 14

Observation 3. Being indoors on screens is perceived as being comfortable and safe, while being outdoors in nature is perceived as uncomfortable and associated with a loss of control

The discomforts associated with being outdoors, whether they are its lack of order, or dealing with weather and the elements, or even threats to physical safety, are additional barriers to these desires to connect to nature. For some, being outdoors also relates to feelings of anxiety whereas being indoors provides an experience of greater comfort and control over one’s environment. This desire for experiences of comfort and feelings of control further contribute to choices to disconnect from nature, and make it easier, or “more appealing”, to (for example) come inside and play video games:

“In a house there is a certain degree of what you think is clean. But when you go outside it is dirty and it is mud. It is not comfortable always when you are outside. But I see why people....it is way more appealing to come inside on your couch and play video games. You don’t have to move or feel uncomfortable or cold or anything like that.” Female, age 14

“I feel safer inside than I do outside. My anxiety level goes down. When I am outside it is hot and all that and it kind of brings me down a little bit. But then inside you can control your environment. If your dog is barking, then put your dog outside and it does not bother you. Or if it is too hot turn on your air conditioning and you can control the environment to make it something that you enjoy.” Female, age 14

Observation 4. The benefits of being in nature became more obvious to young people once they chose to, or were forced to, disconnect from electronic screens

It appeared that young people were making intentional choices related to electronic screen technology that separated them from nature. However, when they found themselves in a situation in which they were forced to disconnect, they realized that nature had some value:

“Me personally I don’t really like nature. But then it is like I feel that because this generation is always on their phone and recently I went on a trip that was isolated from the city. And there were no connections with phones and it was just me and nature. So I kind of got more into nature and like it after that. Once I got back I realized what surrounded me.” Male, age 13

“I am always on my phone when I am home. My mom always says that I am addicted to it and she always will take it from me. But when I was there we were 6 hours from a city and we were in the middle of the woods. Wi-Fi was down and we were on hikes and played sports outside even though we were in grades 10 to 12. We were out more…. The first day was tough because everyone was trying to find internet or phone service. The second day was more relieving. We went out and had sports with other schools and it was easier... We got more into nature. We were taking walks and hiking. So I feel that was a good time for everyone to see an eye opening moment because we were surrounded by forests and it was eye opening.” Female, age 15

### Integration

Four investigators (VM, IJ, NK, WP) participated in this second integration strategy, which was the “triangulation protocol” that involved developing a convergence coding matrix (see Appendix) (Moran-Ellis et al. 2006). When findings from the quantitative and qualitative strands were combined, 23 distinct “observations” emerged (15 quantitative, 8 qualitative), and of these, 13/23 (8 qualitative, 5 from both strands) related directly to our study thread (that high screen levels are in part responsible for declines in the importance of nature in the lives of young people). To foster consensus, after discussion and resolution of the meaning of the words used to describe each observation, we achieved 100% agreement among researchers in the convergence coding of the 23 observations. Agreement or partial agreement between the two strands was reported for 33% (8/23) of these observations, while silence in at least one strand was observed for the 67% (15/23) that remained. While we observed no divergence or disagreement between strands, the use of methodologically specific language led to discussion about the nuances of many observations. The quantitative strand allowed us to observe when consistent patterns were present by age and sex/gender, and also when these patterns differed with respect to the importance of nature and the use of electronic screen technology. It also provided estimates of screen-time use, and the percentages of young people rating connections to nature as important, which could not be captured with qualitative data. In turn, the qualitative strand generated relatively fewer observations, yet it did provide insights into causes and meanings of associations that could not be gleaned from cross-sectional quantitative analyses, including those surrounding the choices being made by young people, the addictive properties of screens, and (dis)comfort at being in outdoor vs. indoor environments.

**Fig. 5:**
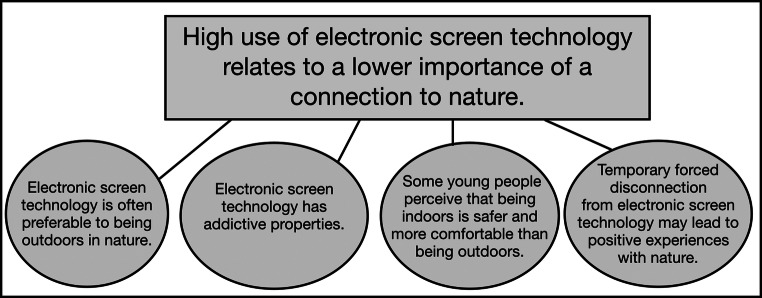
Themes (meta-inferences) identified following integration of qualitative and quantitative strands of this mixed method study

Themes (meta-inferences) emerged from examining both strands simultaneously and are summarized in Fig. 5 (note, these are essentially identical to our four qualitative observations). The themes were as follows: (1) Electronic screen technology is often considered preferable to being outdoors in nature; (2) our study findings speak to the addictive qualities of electronic screen technology in the lives of young people; (3) some young people perceive that being indoors is safer and more comfortable than being outdoors; (4) temporary forced or chosen disconnection from electronic screen technology may lead to positive experiences with nature. These were reached through discussion, debate, and finally consensus as we applied the convergence coding matrix. While it is possible that additional themes could be inferred from the other observations, our consensus was that these did not relate directly to our study thread and associated hypothesis.

## Discussion

In this national mixed methods study, we investigated the idea that exposures to electronic screen technology may be related to declines in the importance of nature in the lives of young people. In addition to using quantitative cross-sectional data from a representative sample to observe patterns and trends surrounding the use of these technologies and their association with connections to nature, the rich qualitative data helped us understand potential explanations for their association(s). While both qualitative and quantitative samples consist of Canadian young people, our study adds to the broader literature of the health impact of screen time in like populations beyond Canadian borders. Major observations that emerged surrounded preferences and choices young people made on how they spent their free time, the possibility of addiction, perceptions of safety and elements of control in indoor vs. outdoor environments, and the recognition by young people who disconnect from electronic screens that such disconnections may heighten one’s appreciation of being outdoors.

Our integrated findings are important for public health efforts aimed at Canadian adolescents. First, we examine one of many possible (perhaps unintended) effects on the lives of young people—that these populations are being denied the potential health benefits of a simple practice (getting outdoors) that has traditionally been inherent to life and well-being of youth during their formative years (Louv 2016). Second, we show that these associations have potential causes, although the latter require confirmation in longitudinal analyses. And third, we provide evidence that this is not a situation without potential for positive change, as young people who choose or are forced to disconnect from screens, even temporarily, recognized the inherent goodness of such choices to their health and well-being.

An existing idea called the “displacement hypothesis” (Subrahmanyam and Šmahel 2011) provides insight into these main findings. This hypothesis proposes that time spent with electronic screens displaces time that young people historically spent engaging in other activities, including going outside, and such practices ultimately have important impacts on health. While use of electronic screen technology may well have some positive benefits, such as efficiencies in engagement in school work and facilitating connections with friends and family (Freeman 2016), many of the behaviours that screen use has displaced represent lost opportunities to foster health. The latter include the loss of physical activity and increase in sedentary behaviour (Gray et al. 2015) (and the ultimate prevention of chronic disease) (Carson et al. 2016) associated with being outside, as well as the social benefits of face-to-face connections with others in natural settings (Brussoni et al. 2015). This theory also speaks to the addictive properties of electronic screen technology. Addictive behaviours are characterized by “overindulgence, tolerance, withdrawal, craving, and loss of control” (Widyanto and McMurran 2004, p. 449). In the case of Internet addiction, evidence suggests that the intrinsic (i.e., internal feelings) more than extrinsic rewards that are associated with online activities increase the likelihood that a person will seek continued and repeated exposure (Chin-Sheng and Chiou 2007). Given our integrative findings, young people may be especially vulnerable to developing addictive behaviours. When this occurs, the resultant choice may be to stay continually connected to others at the expense of being less connected with nature. This tradeoff is described in our qualitative strand as “[taking] away from being outside” (male, age 15) or as something that you “get so caught up in” (female, age 14). By increasingly making choices to stay indoors on their screens, young people are missing out on potential health benefits, such as those associated with coping. The latter include improvements in attention span, self-discipline, coping and healing, and resiliency (Kuo et al. 2019; Brooks et al. 2018; Piccininni et al. 2018; Brussoni et al. 2012). Efforts to disconnect from screens, even temporarily, and engage in these many benefits surely form the basis for efficacious public health and clinical interventions.

The perceived safety and comfort that young people felt when indoors emerged as recurrent observations in the qualitative strand. Many of the young people reported that they simply preferred being indoors on screens than being outdoors in nature. As young people spend more time indoors on screens, and less time outdoors in nature, outdoor environments become less familiar. This may explain why some participants in the qualitative stream described how it was easier to “stay inside on screens” than go outside in nature because of the sense that inside is more comfortable and, to some, feels safer. It is easier to control one’s environment inside than outside. This begs questions around why some young people feel that nature is not a safe environment, and why, when they are out in nature even for short periods, they experience feelings of unease and loss of control. Possible hypotheses for such phenomena include adult messaging surrounding the perceived safety of outdoor spaces (Carver et al. 2008) and a simple lack of familiarity of what it is like to be in a natural yet uncontrolled environment. Such explanations require in-depth study as possible mechanisms that are driving the propensity for young people to choose to stay indoors and hence engaged in primarily sedentary activities.

The gendered findings in this study warrant comment. As described in Fig. 4, when we examined the frequency of exposure to texting, social media use, and instant messaging, we identified strong relationships between electronic communication use and declines in the importance of nature among girls, a pattern that did not appear to be the same for boys. We think it is important that this gendered difference is only present in our findings that relate to the kinds of electronic screen technologies that are used for communication, including to peers and friends (i.e., texting, social media use, and instant messaging). Boys and girls may not only be using these specific kinds of technologies differently but also experiencing different impacts on their mental health. Illustratively, Kelly et al. (2018) have demonstrated that social media use in particular has a greater effect on girls’ mental health than on boys'. These different patterns of usage and of mental health impact may also explain the stronger relationship between social media use and connection to nature in girls.

Methodologically, our intentional use of a mixed methods design was a notable strength of this study. While our cross-sectional observational findings from the HBSC survey (Freeman 2016) provide a national portrait of the extent of engagement of young people with electronic screens, and how this relates etiologically to the importance of nature in their lives, the incorporation of a qualitative strand captures many nuances surrounding this story. We followed two standard strategies for the mixing of evidence from qualitative and quantitative strands, the first being the “following a thread” (Moran-Ellis et al. 2006) concept back and forth between different types of evidence, and the second being use of a “triangulation protocol” (Farmer et al. 2006) in order to maximize the depth of insights from both strands. Adherence to such methods enhances the completeness, depth and rigour of its eventual conclusions. To illustrate, while upon integration we were initially surprised by the high percentage (67%) of findings where one strand was “silent” (O’Cathain et al. 2010), this also points to how much of the story would be missing if one were reliant on only one source of data in isolation. It was also noteworthy that after discussion, there was never any discordance between the four raters, and agreement between the two strands was 100% when not placed into the “silence” category.

Limitations of analysis also warrant comment. These primarily include the limitations of cross-sectional analyses to identify temporal findings requisite for causal inference, and our reliance on secondary data in both strands to explore a novel idea as best we could, yet without the more complete evidence base that would have emerged should electronic screen technologies and the importance of nature have been the primary focus of the research initiative that spawned this analysis. As next research steps, the observations and themes (meta-inferences) identified require confirmation in investigations, both qualitative and quantitative, where the relationship between electronic screen technology and the importance of nature becomes the primary research focus. In addition, the triangulated results require formal review with young people, educators, parents, and health professionals to fully complete the triangulation protocol developed by Farmer et al. (2006).

Should the study findings be confirmed, they do point to actionable implications for applied public health practice. First, we require sensible public health initiatives that work to intentionally reframe adult-driven fears about being in unprotected environments, and hence perceived danger of being connected with nature. Second, all those who are involved in the education and supervision of children could be intentional in teaching young people how best to be comfortable in outdoor environments. Third, our qualitative findings surrounding childhood experiences of “forced disconnection” led our study participants to a greater appreciation of and connection to nature. One way of mitigating the barriers that electronic screen technology pose to connections to nature is very simply to provide intentional opportunities to disconnect for a period of time. Each of these suggestions will require understanding and commitment on the part of young people, and the adults responsible for their care and nurturing.

## Appendix

**Table:** Convergent coding matrix

Meta-inferences (themes)		Convergence between strands			
Key observations (obtained from one or both of strands 1 and 2)		Agreement		Silence	Dissonance
		Full	Partial		
Theme 1. Electronic screen technology is often preferable to being outdoors in nature					
1.	Young people prioritize being indoors over being outdoors			X (2)	
2.	Many youth report liking the outdoors, but it does not appear to be as important as screens		X		
3.	Screen use is negatively associated with connections with nature	X			
Theme 2. Electronic screen technology has addictive properties					
4.	Young people are in front of screens a great portion of their days	X			
5.	Screen-time levels are very high	X			
6.	Addictive property of screens interferes with nature connection			X (2)	
Theme 3. Some young people perceive that being indoors is safer and more comfortable than being outdoors					
7.	It is more “comfortable” to be on screens indoors than to be outdoors			X (2)	
8.	It feels “safer” to be on screens indoors than to be outdoors			X (2)	
9.	Being outdoors comes with feelings of “loss of control”			X (2)	
10.	Being outdoors comes with feelings of “loss of safety”			X (2)	
Theme 4. Temporary forced disconnection from electronic screen technology may lead to positive experiences with nature					
11.	Many young people value connections to nature as important or very important		X		
12.	Young people recognize the benefits of exposures to nature, at least conceptually			X (2)	
13.	Temporary disconnection led to increased reconnection to nature			X (2)	
Other observations (e.g., patterns observed by age, gender, and type of electronic screen technology)					
14.	Screen time increased as participants got older	X			
15.	Perceptions that nature is important decreased as children got older	X			
16.	Social media/texting related to nature connections in girls only			X (1)	
17.	Social media/text/IM are more common in girls than boys			X (1)	
18.	Social media/text/IM are more common in older children			X (1)	
19.	Use of cell phones is more common in older children			X (1)	
20.	There seem to be differences between association with tech use and nature depending on tech use (whether using to communicate with friends vs. total screen time)			X (1)	
21.	Negative association between screens and disconnection to nature is weaker in older children			X (1)	
22.	Nature appears to be slightly more important to girls than to boys			X (1)	
23.	Boys are using video games (TV and movies) more than girls			X (1)	

(1) Qualitative (strand 1) provides no information (it is “silent”) on the key observation

(2) Quantitative (strand 2) is “silent” on the key observation
